# Spastic Paraparesis Following Cocaine Inhalation

**DOI:** 10.1155/2009/465968

**Published:** 2009-05-04

**Authors:** Henry Oluwasefunmi Savage, Malin Roesner, David Cohen

**Affiliations:** ^1^Cardiology Department, Royal Brompton Hospital, Sydney Street, London SW3 6NP, UK; ^2^Department of Elderly Care Medicine, Northwick Park Hospital, Watford Road, Harrow Middlesex HA1 3UJ, UK

## Abstract

Cocaine use is reaching epidemic proportions in the UK and the consequences are a number of debilitating effects. Strokes may result from a number of mechanisms related to cocaine use. This report describes a case of cocaine induced stroke in an apparently healthy young man with unusual patterns of radiological findings on his brain MRI.

## 1. Introduction

Cocaine dependence is an enormous public health problem due to its vast array of medical complications. Cerebral ischaemia is a recognized neurovascular effect of cocaine use. We report the case of a young gentleman who presented with spastic paraparesis shortly after cocaine inhalation and an unusual pattern of ischaemia seen on magnetic resonance imaging of his brain.

## 2. Case Report

A 27-year-old male Caucasian presented with sudden onset paraparesis. He had been on a social night out and admitted to nasal inhalation of small quantities of cocaine about an hour before onset of symptoms. There was no significant past medical history of note, and he took no regular medication. He however admitted to recreational consumption of cocaine for a few years.

Clinical examination revealed a regular heart rate of 90 beats per minute and an elevated blood pressure of 148/70 mmHg. A 12-lead ECG performed was normal sinus rhythm with no evidence of ischaemia. He had a right Horner sign, and there was grade1 power in both legs with increased tone and brisk reflexes. Babinski sign was positive bilaterally. Sensory examination was normal. The rest of his cranial nerve examination was unremarkable. His Glasgow coma scale was 14/15, losing 1 verbal point due to his mild confusion. He was however of normal GCS by the following day, 12 hours later.

**Figure 1:**
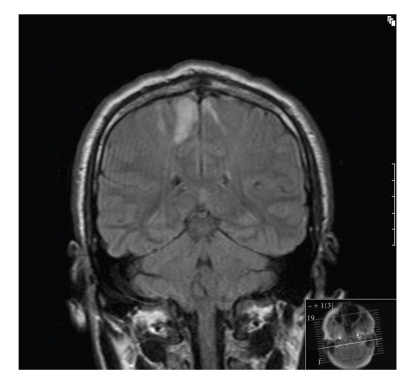
T1/T2 weighted MRI images showing parasagittal infarcts.

Because of his clinical presentation, an urgent magnetic resonance image of his spine was performed initially which surprisingly did not reveal any abnormality. As a consequence, the radiologist on discussion with the medical team proceeded to MRI of his brain which demonstrated extensive subcortical white matter parasagittal cerebral infarcts with focal lesions seen involving the globus pallidus bilaterally, corpus callosum, and the right cerebellar hemisphere (See Figure 1). A urine sample was positive for opiates and cocaine. Our patient categorically denied opiod use, and the team agreed that this may have been a false positive test.

Full blood count and electrolytes were unremarkable and an autoimmune screen was negative. His fasting blood sugar and lipid profile was normal. Cerebral arteriography and venography were normal. Echocardiography demonstrated a patent foramen ovale; however, doppler ultrasound of the deep veins of his legs revealed no thrombosis. A 12-lead electrocardiograph revealed sinus rhythm.

He was managed on our stroke unit, receiving intensive neurorehabilitation and regaining mobility with crutches after 12 days. A cardiology review concluded that there were no compelling indications for closure of his atrial septal defect in view of a probable cause of his stroke. His blood pressure throughout his admission was normal. He was thus discharged home after 2 weeks of inpatient stay with a referral to his local drug and alcohol abuse service. He has so far made a complete recovery.

## 3. Discussion

Cocaine remains a common non alcoholic drug of abuse in the United Kingdom, and this seems to be on the rise. Its use along with other drugs of abuse continues to pose a serious public health problem. There were 162 203 drug users in contact with drug treatment services and GPs for 2008 in the UK [1].

The UK has the highest adult prevalence of both lifetime and recent cocaine use in Europe, and levels of use among younger adults and people who lived in urban areas tended to be higher than the population average [2]. The most recent data available puts the lifetime prevalence of cocaine use for the UK adult population at 7.7% (i.e., have used at least once). The range of lifetime experience among European 15- to 34-year-olds is between 0.4% and 11.2%, and the UK is also at the top of this range [2].

Acute cocaine toxicity may result in ischaemic or hemorrhagic strokes. A variety of neuropsychiatric complications are also well recognized [3, 4].

Cocaine is thought to cause strokes through various proposed mechanisms. This includes global cerebrovasospasm as a result of its potent vasoconstrictor ability which may lead to global cerebral hypo perfusion. Dose-related cerebral vasoconstriction on magnetic resonance angiograms has been observed [5]. Cocaine prevents the reuptake of noradrenaline, serotonin and dopamine at pre-synaptic nerve terminals. It may also act directly on the vascular smooth muscle cells by direct effects on calcium channels, promoting intracellular calcium release from the sarcoplasmic reticulum. There is post mortem evidence to support vasospasm with consistent histological findings in nasal arterioles [6], intestinal arterioles [7], and coronary arteries [8].

Cocaine may disrupt cerebral auto-regulation and is found to reduce cerebral blood flow in users as found by Volkow et al. [9] and a Californian group [10]. There is also evidence to suggest reduced metabolism following cocaine administration which may also result in down regulating cerebral blood flow [11].

Atherosclerosis is an important consequence of cocaine use, and this has been demonstrated in the cerebral vasculature. The mechanism of atherosclerosis is thought to relate to vascular injury from vasospasm leading to endothelial injury and the consequence of thrombus formation. Increased platelet aggregation may also play a part.

In vivo studies have demonstrated increased platelet aggregation secondary to cocaine administration [12]. This mechanism of causing strokes is important because even in the absence of atherosclerotic disease or vascular endothelial damage thrombus formation is possible.

An inflammatory vasculopathy may result from cocaine use but this is less common. The basis of cerebral vasculitis has been attributed to angiographic findings of vessel necrosis and arterial beading [13].

Cardioembolism is another important cause of strokes from embolised impurities either in patients who utilize the intravenous route or in those who suffer cocaine-related MI and arrhythmias or develop cardiomyopathies [14]. It is important to continue to follow these patients up.

The extensive and symmetrical pattern of subcortical infarcts noted in the parasagittal region in our patients MRI is an unusual finding, and these lesions were clearly related to their dramatic symptoms at presentation.

In view of his presentation immediately post inhalation of the drug and mild abnormality of his GCS, we theorised that our patient may have been a victim of sudden onset global cerebrovasospasm. This, we believed, resulted in focal damage to both large and small cerebral vessels.

It is conceivable to associate the pattern of injury in our patient to the variety of proposed mechanisms of cocaine-related cerebrovascular injury. The possibility of cardioembolism via his patent foramen ovale remained present; however, our patient-denied intravenous use of drugs and certainly did not have any obvious clinical signs to the contrary. Other investigations were unable to conclusively identify any other mechanisms that may have been contributory, even though our patient had radiological evidence of some diffuse disease.

We monitored his blood pressure through admission which was consistently normal and thus did not treat for hypertension. On discharge, we recommended that his general practitioner follow this up. There were no other risk factors for cerebrovascular disease identified.

In conclusion, with the seemingly upward trend in cocaine consumption in the UK, it is important to recognize the possible presentations of cocaine-induced cerebrovascular disease. It remains necessary to identify other risk factors for cerebrovascular disease in these patients. Ultimately, making a prompt diagnosis and offering such patients immediate treatment and long-term management in terms of counselling and contact with drug treatment services may improve their lives.
